# Barriers to the Implementation of Max-Profit and Stochastic Feed Formulation Strategies: A Survey of the Australian Poultry Industry

**DOI:** 10.3390/ani14223333

**Published:** 2024-11-19

**Authors:** Amy Moss, Anh Chung, Hiep Dao, Greg Parkinson, Tamsyn Crowley

**Affiliations:** 1 School of Environmental and Rural Science, University of New England, Armidale, NSW 2351, Australia; tchung4@une.edu.au (A.C.); tdao2@une.edu.au (H.D.); 2 Livorno Consulting, Brunswick, VIC 3056, Australia; g.parkinson@iinet.net.au; 3 School of Medicine, Deakin University, Geelong, VIC 3220, Australia; tamsyn.crowley@une.edu.au

**Keywords:** stochastic, max-profit, feed formulation, survey, poultry, diets

## Abstract

A survey of industry groups including nutritionists, producers, feed manufacturers, and technical personnel in broiler and layer operations within the Australian poultry industry was completed to determine the industry’s present views of max-profit and stochastic feed formulation and the barriers they see to implementing these techniques. A total of 32 responses were collected, made up of 17 nutritionists, 4 feed manufacturers, 5 producers, and 6 technical personnel. The survey revealed interest and need to implement stochastic and max-profit feed formulation techniques and identified the key barriers to the implementation of these feed formulation techniques. Barriers identified included limited software to assist nutritionists in using these feed formulation techniques, less data collection than in other animal industries, insufficient training, and possible restrictions on nutritionists via KPIs to minimise diet cost. It was identified that layer farms did not routinely use near-infrared spectroscopy (NIRS) to estimate the nutrient content of feed ingredients and so mostly relied on book values and historical data. Thus, stochastic feed formulation may be particularly useful to buffer the uncertainty and ensure the minimum nutrient requirements of flocks are met. Recommendations were drawn to improve the adoption of max-profit and stochastic feed formulations of Australian layer diets.

## Simple Summary

Max-profit and stochastic feed formulation are alternative approaches to least-cost feed formulation that have been known for many years; however, despite the potential benefits, they are seldom used in the poultry industry as a tool to formulate diets. Therefore, the authors completed a survey of the Australian poultry industry to determine the barriers to their use. Barriers identified included limited software to assist nutritionists in using these feed formulation techniques, less data collection than in other animal industries, insufficient training, and possible restrictions on nutritionists via KPIs to minimise diet cost. While there are barriers to the implementation of max-profit and stochastic approaches, there are many opportunities to be gained in these areas to reduce the variability of the nutrient content of diets for poultry, improve tools to inform decision-making, and enhance the profitability and sustainability of the Australian poultry industry. Recommendations to overcome these barriers and challenges are discussed.

## 1. Introduction

Increased flexibility in feed formulation via alternative approaches to the least-cost feed formulation model may bring economic and production advantages for the poultry industry, as demonstrated in Moss et al. [1] and Pesti and Seila [2]. The concept of alternative approaches to least-cost feed formulation, such as max-profit feed formulation or stochastic feed formulation, has been known for many years. For example, Almquist [3] demonstrated the advantages of implementing the law of diminishing returns and economic principles to feed formulation some 70 years ago. More modern demonstrations also report the benefits of employing economic principles for least-cost feed formulation [1,4]. Nevertheless, despite these alternative techniques and their advantages being known for some time, the use of these techniques within the Australian poultry industry is undetermined but is likely low, given the emphasis on least-cost feed formulation.

Traditionally, least-cost feed formulation has been a practical approach to solving feed formulation problems, particularly before computers became as powerful and accessible as they are today. Simple linear programs allowed nutritionists to easily solve the least-cost feed formulations [5]. Furthermore, the data collection and analysis required to perform stochastic and economic calculations may also have been hindered before modern computing and the development and collection of ‘big data’ within poultry operations [6]. However, with the current development of smart farming and precision agriculture technologies, poultry nutritionists may now have more access to data than ever before. This pertains not only to production and market data but also data on feed ingredients such as NIRS and a growing number of databases offered by commercial companies and organisations, which summarise not only the mean nutrient values of common ingredients but the standard deviation for reported nutrients as well [7]. This brings many opportunities but also complications, such as data acquisition, storage, analysis, and the generation of meaningful recommendations [6].

Within the rigid framework of least-cost feed formulation, there may not be an opportunity to input or employ big data for maximum benefit. Thus, least-cost feed formulation has disadvantages as it may not necessarily represent the most economical solution. Furthermore, the nutrient content within feed ingredients is variable and can lead to significant economic losses if this is not accounted for or addressed in some way [8]. Within least-cost feed formulation, nutritionists must estimate matrix values to build a safety margin to account for this uncertainty, as this method of feed formulation has no inbuilt mechanisms to deal with this variability. Hence, analysing different scenarios and minimising the probability that the feed may not meet minimum nutrient requirements are difficult.

Feed formulation is not just an exercise in nutrition or animal biology but also one of economics, as poultry operations are commercial enterprises. The main aim of any commercial enterprise is usually to maximise profits with the resources or inputs available. To maximise profits within a business, it is necessary to hold a firm understanding of the cost of inputs and the value of outputs and to model the relationship between the two to determine the maximum profit achievable. Unfortunately, the least-cost diet formulation is limited as it does not take this relationship into account and, thereby, does not necessarily generate the optimal solution to maximise profits [9]. This not only restricts the profitability of a poultry operation but also the flexibility and ability to cope with economic and market fluctuations, which have become commonplace in Australia and globally in current times.

Furthermore, due to the nature of variability within feed ingredients and the practicalities of thoroughly sampling every shipment of ingredients coming into the mill and every tonne of feed leaving the mill, uncertainty also exists within feed formulation. This is particularly difficult within the Australian layer industry due to its non-integrated nature [10]. Stochastic feed formulation is an optimisation procedure based on probability distribution and is used in situations of high uncertainty, such as nutrient variation in feedstuffs [11]. Thus, stochastic feed formulation, which allows the formulation of diets that meet a specified probability of attaining the required nutrient minimums, may assist nutritionists in managing this uncertainty and risk. Thus, this technique may have direct practical application in poultry feed formulation.

Despite the advantages of max-profit and stochastic feed formulation, the likelihood is that it is not commonly used within the Australian poultry industries. In order for producers to accurately formulate diets using max-profit and stochastic techniques, it is possible that data are presently lacking, and there may be some barriers to adoption. For example, in the collection and management of production and market data, the need for hen’s response and performance models, further training, or more sophisticated economic modelling tools may be required to ensure adoption. Therefore, this study aimed to determine the poultry industry’s present views of max-profit and stochastic feed formulation and the barriers it sees to implementing these techniques. This was explored via a survey of various industry groups, including nutritionists, producers, feed manufacturers, and personnel undertaking technical roles in broiler and layer operations.

## 2. Materials and Methods

This survey was approved by the Human Research Ethics Committee within the University of New England (approval number HE21-122). An online survey was developed and distributed via Survey Monkey Inc. (© 2021) (San Mateo, CA, USA) to determine the current use of max-profit and stochastic feed formulation techniques within the Australian poultry industry and then, if these techniques were not used by the respondent, determine the barriers to future use. A questionnaire was prepared (Table 1), and the survey was distributed via a link provided in various Australian poultry newspapers, newsletters, and industry webinars to reach many different sectors within the Australian poultry industry. Responses were anonymous and collected between June and August 2021. A total of 32 responses were collected and included 17 nutritionists, 4 feed manufacturers, 5 producers, and 6 technical personnel. Despite the small number of respondents, this survey was responded to by the vast majority of poultry nutritionists within the country. The Australian poultry industry is relatively small scale; for example, for broiler production, only two companies hold the majority market share (>80%), which employ only a few nutritionists each. Thus, the small number of responses collected is still relevant and meaningful for this evaluation.

**Figure 1:**
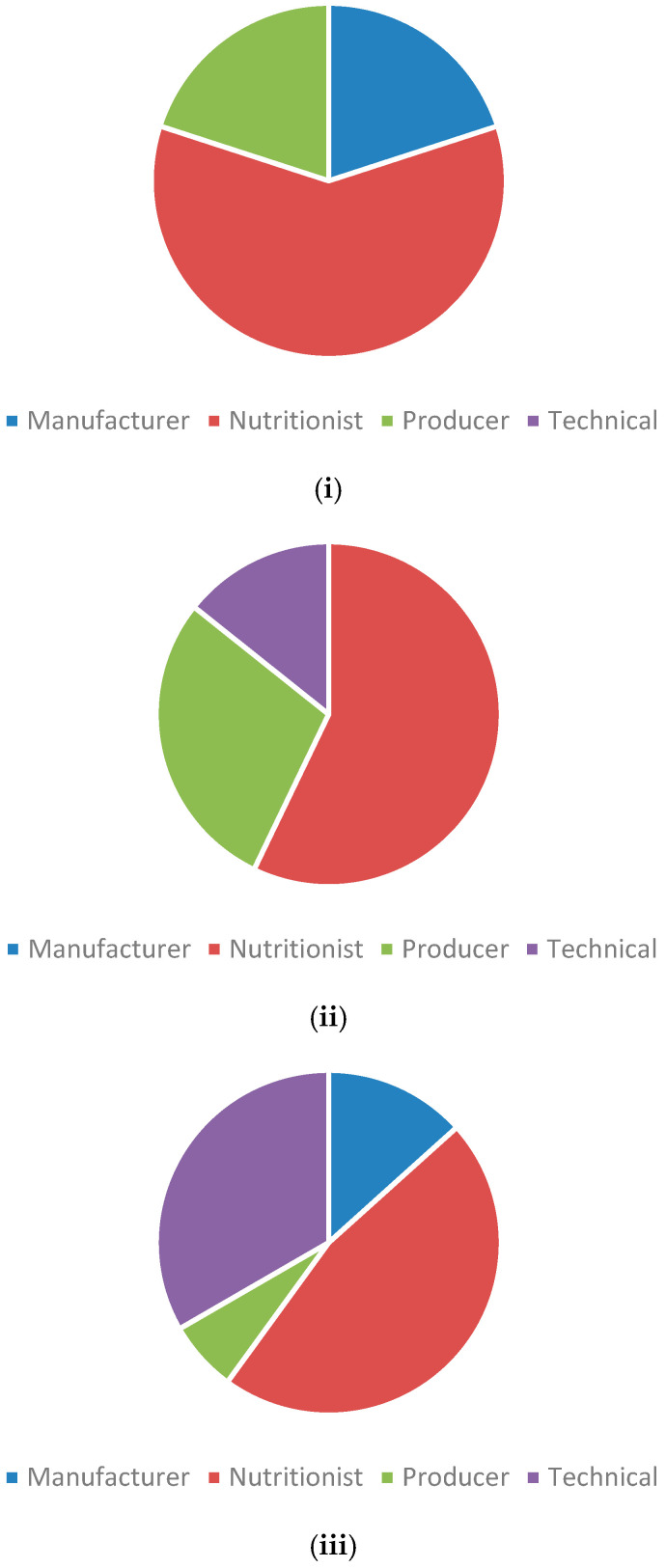
Distribution of job roles of respondents within the layer industry (**i**), broiler industry (**ii**), and those who evenly work for both (**iii**).

Of the respondents, 10 were employed in large integrated companies, while the majority (22) were employed within stand-alone/independent businesses. Of the respondents, 15 worked in both the broiler and layer industry, 7 within the broiler industry alone, and 10 within the layer industry alone. The vast majority (80%) of respondents who identified as working within the layer industry or both layer and broiler industries were employed within stand-alone/independent businesses, whereas the vast majority of respondents who identified as working within the broiler industry (70%) were employed in an integrated company. Among those who responded, there was a fairly even distribution of job roles within both the broiler and layer industries (Figure 1).

**Figure 2:**
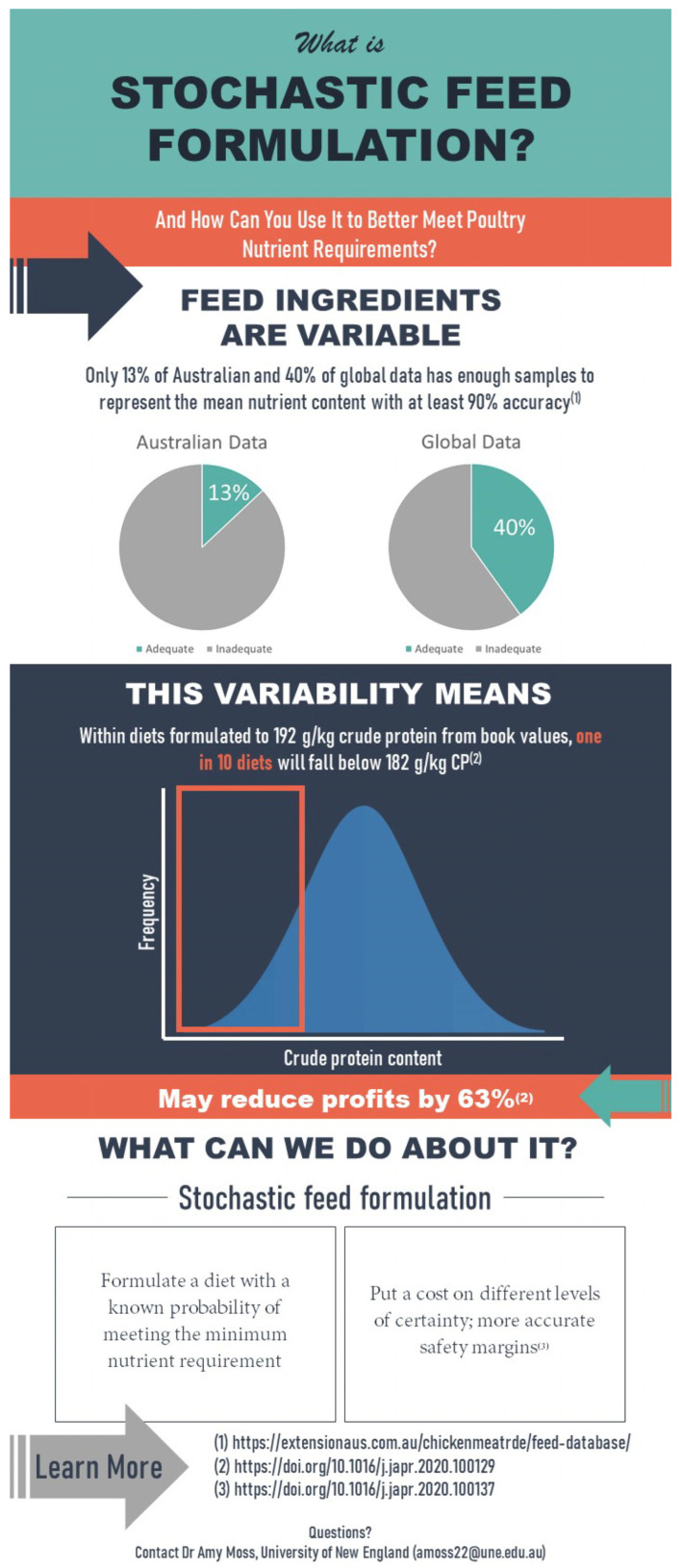
Stochastic feed formulation infographic was shown to respondents following question 10 of the survey to ensure they had the information required to answer questions 11 and 12.

**Figure 3:**
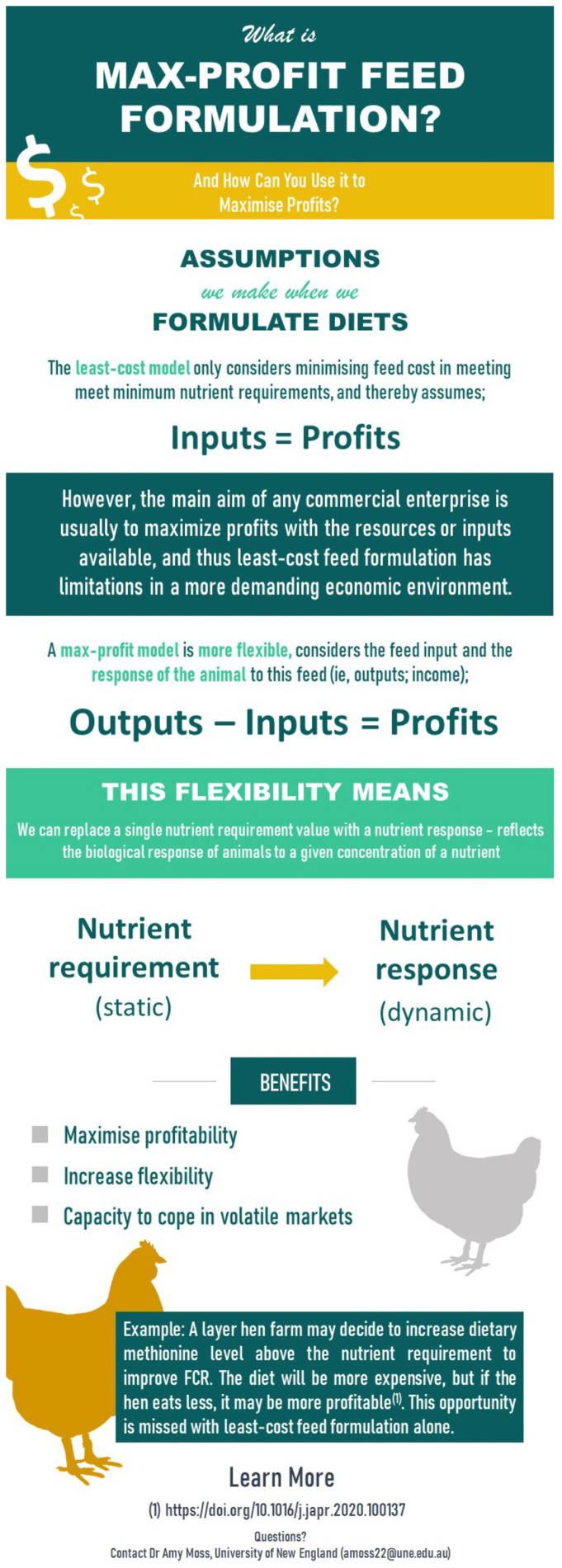
Max-profit feed formulation infographic was shown to respondents following question 13 of the survey to ensure they had the information required to answer questions 14 and 15.

Following the demographic information and questions regarding NIR and feed variability, the survey then asked respondents if they were aware of stochastic feed formulation, if they formulate diets, and whether they used stochastic feed formulation. As not all respondents may know what this is, they were then shown an infographic (Figure 2) to explain what stochastic feed formulation was so they could answer the subsequent questions regarding whether they would be interested in using it if they had further resources. Next, respondents were asked the same questions regarding max-profit feed formulation and again shown an infographic (Figure 3) in case they were not aware so they could answer the subsequent questions pertaining to max-profit feed formulation.

The data were checked, and no data were excluded from the final data set. The data were exported from Survey Monkey and analysed via frequency counts, proportions, and descriptive statistics approaches in IBM SPSS statistics program version 29 (IBM Corporation, Somers, NY, USA).

## 3. Results

The preference of data source for the nutrient content of feed ingredients for feed formulation is shown in Table 2. It is evident that layer nutritionists prefer book values or historical data, while broiler nutritionists almost solely prefer NIR. When asked if NIR was routinely used, 60% of respondents in the layer industry said yes, in comparison to 85.7% and 66.7% of respondents in the broiler industry and both industries, respectively. The main reason for not using an NIR service was listed as cost, followed by practicality. The accuracy and reliability of NIR services were also frequently cited as reasons not to use these services.

The number and proportion of yes/no responses to questions regarding various aspects of feed formulation are given in Table 3. When asked if respondents were concerned about the variability of feed ingredients, 84.4% answered yes. Those who were not concerned were evenly spread between the broiler and layer industries, as well as integrated and stand-alone companies. When asked if respondents were concerned about whether the variability of feed ingredients impacts the feed that reaches the farm, 90.6% were concerned.

A total of 78% of respondents were aware of stochastic feed formulation, but only 12.5% used stochastic feed formulation. A total of 87.5% of respondents were aware of max-profit feed formulation, but 28.1% used max-profit feed formulation.

The number and proportion of those who would require the following supports to start using stochastic and max profit feed formulation is given in Table 4. Stochastic feed formulation would be used if respondents had access to the following supports: training and more information (38%), stochastic feed formulation software (32%), and better data on the variability of ingredients (24%). A total of 6% would not use it no matter the support provided. One respondent commented that as they are a small operation, they do not have the batch sizes sufficient so as to receive any real advantage from stochastic feed formulation. Max-profit feed formulation would be used if respondents had access to the following supports: training and more information (29.5%), max-profit feed formulation software (31.8%), and better data collection (31.8%). The remaining 6.8% would not use max-profit feed formulation.

## 4. Discussion

Almost 50 years prior to this survey, Lerman and Bie [12] concluded that feed ingredient variability was of concern for the intensive animal industries, and perhaps the main cause of this variability is improper sampling. Since this report, the variability of Australian feed ingredients has recently been highlighted [7], where it is evident that the variation reported in the nutrient contents of many ingredients in Australian datasets is even greater than the variation reported in the same ingredients in global datasets. This highlights the variability that can exist within Australian feed ingredients (where the main cereals used consist of wheat and sorghum and the main oilseed meals are imported soybean meal and locally sourced canola meal) and is likely due to the broad range of growing conditions and production methods across our vast country, as it has been demonstrated that origin has a large impact on the nutrient content of feed grains [13]. Thus, it makes sense that in the present survey, the industry has conveyed concern regarding the variability of our feed ingredients. Grain Trade Australia [14], which sets the national guidelines for sampling, has stated that the studies behind their recommendations were conducted many years ago and that variability does exist between probe types. Whilst the focus is often on laboratory error when analysing a sample, it is estimated that sampling error may actually be 100 times that of the analytical error [15]. Therefore, the sampling procedures we use hold great importance. But unfortunately, this issue has not been made a priority despite the concern surrounding ingredient variability within the industry. Furthermore, [8] demonstrates that sampling variability is costly to the Australian poultry industry. Therefore, improving the sampling methodology should be a research priority. As stochastic feed formulation can take the variability of nutrients in ingredients into account in feed formulation [1], it should be helpful to reduce the impact of variability in Australian feed ingredients.

When considering feed formulation needs for the Australian poultry industry, it is important to take into account the two main structures within the industry: independent and integrated businesses. Within the survey, the majority of respondents who identified as working within the layer industry or both layer and broiler industries were employed within stand-alone/independent businesses, whereas the vast majority of respondents who identified as working within the broiler industry were employed in an integrated company. This is to be expected, as it reflects the current situation within the Australian poultry industry where broiler companies are integrated with their own feed mills, breeder farms, and hatcheries, while layer farms are usually owned by the individuals, lack integration [16], and buy their feed and pullets from separate suppliers [10,17]. Thus, there are distinct organisational structural differences, each with differing needs and perspectives for farm design and management practices [18], but also for feed formulation, feed manufacturing, and production. For example, the survey reported that much fewer of the layer industry’s producers use NIR as compared to the broiler industry, as they are typically not integrated, and the cost and practicality of using this service would be more prohibitive than for a large integrated company. Thus, stochastic feed formulation may be particularly valuable for the laying hen industry, as the nutrient composition of feed ingredients is largely unknown, and book values are often used.

The major barriers preventing the use of stochastic feed formulation raised in this survey were the need for further training and access to software capable of performing stochastic feed formulation. In the case of max-profit feed formulation, the capacity for better market and production data collection and max-profit feed formulation software would be of advantage. Previously, it was reported that the Australian layer industry has less reporting of physical and financial details than other industries [19]. This is likely due to the non-integrated nature of the Australian layer industry, as there is a high degree of private ownership [18]. Thus, the layer industry, in particular, may benefit from improvements in data collection.

Finally, the structure of integrated companies may present a barrier in itself. One respondent from an integrated company noted that if max-profit feed formulation were to be implemented, internal costings would need to be restructured as the current profit system stops at the processing plant, and presently, the feed conversion ratio (FCR) and cost per bird are all that is accounted for. This is an excellent point, as in Australia and many other countries, integrated broiler operations evaluate their nutritionists by how cheaply they may formulate feed, evaluate their farm managers by their FCR, and evaluate their processing managers by the processing yields. However, by constricting the nutritionist to the cheapest diet possible, this may worsen performance and impact carcass yields, thereby impacting the performance of the farm manager and processing plant manager. Likewise, when restricted to least-cost diets, the feed may not be formulated to minimise wet excreta, and the cost of wet litter is borne by the farm manager. A similar issue may be seen within independent businesses, such as within the laying hen industry. If producers and nutritionists struggle to match the goals of feed formulation with those of the layer enterprise (for example, to meet the demand of a particular market) and do not have sufficient communication to convey how these goals may be achieved (for example, feeding a slightly more expensive diet for the improvement of egg quality), then the profitability of the industry may suffer. Thus, the implementation of max-profit feed formulation may present a good opportunity for such costing and performance evaluation structures to be re-evaluated. This re-evaluation of cost structure may also be required in the implementation of the Emissions Reduction Fund [20] by the Australian Government, which will distribute carbon credits to businesses that reduce emissions. With the current cost structures within the poultry industry, it is unclear how these carbon credits will be distributed.

## 5. Conclusions

In conclusion, there is clear interest and need for the Australian poultry industry to implement stochastic and max-profit feed formulation techniques more broadly. Barriers identified to broader use included limited software to assist nutritionists in using these feed formulation techniques, less data collection than in other animal industries, insufficient training, and possible restrictions on nutritionists via KPIs to minimise diet cost.

Thus, to support this requirement, the authors recommend further training on stochastic feed formulation, software that is capable of stochastic and max-profit feed formulation, and better market and production data collection, as these remain barriers to the adoption of these approaches. Thus, there is a requirement for feed formulators that combine production tools, bird models, economic data, and feed ingredient data in a holistic manner. Such tools may exist to certain extents, but with the range of software on the market, it is difficult to identify what is available. Other key barriers identified to the implementation of these techniques within the industry are the current cost structures and KPIs, which restrict the flexibility of feed formulation and thereby limit the economic sustainability of the industry. Such costing and performance evaluation structures require re-evaluation.

## Figures and Tables

**Table 1:** Survey questions asked of respondents.

Question #	Questions
1	What sector of the industry is your job title best described as?
2	Are you part of an integrated operation or a stand-alone company?
3	Do you primarily work with the broiler or layer industries, or do you spend an equal amount of time with both?
4	Where do you obtain data on the nutrient content of feed ingredients from? Please rank their importance.
5	If you use NIR or book values, which sources/companies do you use?
6	Do you routinely use NIR?
7	If not, what is restricting NIR use? (Select all that apply)
8	Are you concerned about the variability of feed ingredients?
9	Do you think that the variability of feed ingredients impacts the feed that reaches the farm?
10	Are you aware of stochastic feed formulation (i.e., adjusting diets based on the probability of meeting a minimum nutrient requirement)?
11	Do you use stochastic feed formulation?
12	If you do not know/use stochastic feed formulation already, would you start using stochastic feed formulation if you had one or more of the below? (select all that apply)
13	Are you aware of max-profit feed formulation (i.e., adjusting diets beyond the least-cost formulation, taking into account outputs such as the production of eggs to maximise profits)?
14	Do you use max-profit feed formulation?
15	If you do not know/use max-profit feed formulation already, would you start using max-profit feed formulation if you had one or more of the below? (select all that apply)
16	Any additional comments?

**Table 2:** Number of people in each sector (% given in parenthesis) who prefer to use NIR, book values, or historical data, and if NIR was routinely used for feed formulation.

First Choice Between NIR, Book Values, and Historical Values as the Most Important Method for Retrieving Data on Nutrient Value			
Sector	NIR (%)	Book Values (%)	Historical (%)
Broilers	4 (80)	1 (20)	0 (0)
Layers	1 (20)	2 (40)	2 (40)
Both	3 (25)	7 (58.3)	2 (16.7)
**Do you routinely use NIR?**			
**Sector**	Yes	No	
Broilers	6 (85.7)	1 (14.3)	
Layers	6 (60)	4 (40)	
Both	10 (66.7)	5 (33.3)	

**Table 3:** Number and proportion (% given in parentheses) of yes/no responses to questions regarding various aspects of feed formulation.

Question	Yes (%)	No (%)	N/A (%)
Are you concerned about the variability of feed ingredients?	27 (84.4)	5 (15.6)	-
Do you think that the variability of feed ingredients impacts the feed that reaches the farm?	29 (90.6)	3 (9.4)	-
Are you aware of stochastic feed formulation?	25 (78.1)	7 (21.9)	-
Do you use stochastic feed formulation?	4 (12.5)	19 (59.4)	9 (28.1)
Are you aware of max-profit feed formulation?	28 (87.5)	4 (12.5)	-
Do you use max-profit feed formulation?	9 (28.1)	15 (46.9)	8 (25.0)

**Table 4:** Number and proportion (% given in parenthesis; per each formulation method) who would require the following supports to start using stochastic and max profit feed formulation.

Support	Stochastic	Max Profit
Training and more information	19 (38)	13 (29.5)
Feed formulation software with stochastic feed formulation built-in	16 (32)	14 (31.8)
Better data on the variability of feed ingredients you use	12 (24)	14 (31.8)
I would not use it	3 (6)	3 (6.8)

## Data Availability

The data that support this study will be shared upon reasonable request with the corresponding author.
